# Affective reserve in multiple sclerosis

**DOI:** 10.1177/13524585251383381

**Published:** 2025-10-15

**Authors:** David E Freedman, Karen A Campbell, Anthony Feinstein

**Affiliations:** Department of Psychiatry, Sunnybrook Health Sciences Centre, Toronto, ON, Canada; Department of Psychiatry, Temerty Faculty of Medicine, University of Toronto, Toronto, ON, Canada; School of Nursing, Faculty of Health, York University, Toronto, ON, Canada; Department of Psychiatry, Sunnybrook Health Sciences Centre, Toronto, ON, Canada; Department of Psychiatry, Temerty Faculty of Medicine, University of Toronto, Toronto, ON, Canada

**Keywords:** Depression, affective reserve, cognitive reserve, multiple sclerosis, neuropsychology

In a medical model of illness, such as multiple sclerosis (MS), there is a foundational concept that disease activity (e.g. inflammation and neurodegeneration) leads to clinical symptoms. Yet, in people with multiple sclerosis (pwMS), increasing disease pathology is accompanied by variability in cognitive and physical functioning. The identification of this discrepancy contributed to the recognition of “cognitive reserve.”^1^ In Sumowski and Leavitt’s model, high cognitive reserve attenuates the relationship between disease pathology and cognitive functioning, explaining why some pwMS experience widespread brain injury, but do not have cognitive impairment.^1^ Physical reserve, a complementary construct that reflects the buffer between physical decline and reduced motor function, was introduced next.^2^ Investigators have even proposed “individual reserve” to capture combined cognitive and physical reserve.^2^ This developing literature on reserve in MS begs the question, what about affective reserve?

In this theoretical paper, we discuss how current knowledge about depression in pwMS may support the concept of affective reserve. We offer a definition of affective reserve in the context of MS, suggest potential ways to measure the factors that we posit comprise it, and discuss its potential benefits for pwMS, clinicians, and investigators.

## Depression in MS

Depression is a persistent state of prominent sadness or anhedonia, often associated with cognitive and vegetative symptoms, and linked to distress or functional impairment.^3^ This debilitating condition is two to three times more common in MS compared to the general population and contributes to reduced quality of life, worse functional outcomes, and increased mortality among pwMS.^3^

Several biological and psychosocial factors may influence the risk of depression in pwMS. Depression is associated with atrophy in the prefrontal cortex and deep gray matter of the striatum, ventral diencephalon, thalamus, and amygdala.^3,4^ White matter lesions in a transdiagnostic “depression network” involving the medial prefrontal cortex, retrosplenial cortex, intraparietal sulcus, medial temporal lobe, and the ventral tegmental area may also contribute.^5^ In addition to an elevated T2 lesion load, increased depressive symptoms in MS are intertwined with reduced frequency of CD4^+^ CCR7^low^T_CM_ cells.^6^ While the direction of this link is unclear, it emphasizes that immune system dysregulation underlies depressive symptoms in pwMS. Personality traits and coping styles also influence depression risk. Neuroticism, introversion, pessimism, emotional dysregulation, and psychological rigidity may increase vulnerability to depressive symptoms.^7^ Among pwMS, a problem-solving coping style, in contrast to emotion-oriented or escape avoidance-based coping styles, may protect against depressive symptoms.^3^ This understanding of the biopsychosocial contributors to depression in pwMS sets up the rationale for introducing the concept of affective reserve.

Despite an elevated prevalence of depression among pwMS, there are inconsistent weak links between clinical markers of MS disease pathology, such as disease duration or physical disability, and depressive symptoms.^3^ Clinicians frequently encounter patients with substantial disability and a heavy burden of disease on imaging, but who are not depressed. Affective reserve may help to explain the discrepancy between clinical markers of disease pathology and depressive symptoms in MS.

## Defining affective reserve

Integrating existing concepts of affective reserve and resilience in the general population, affective reserve can be characterized as a person’s “capacity to regulate affective states”^8^ (e.g. emotions or moods) to explain the variable responses to biopsychosocial adversity in MS.^8,9^ A focal outcome of interest is whether an individual with MS develops depression. These adversities may include (but are not limited to) disease pathology (often measured with neuroimaging abnormalities), physical disability, MS symptoms, psychosocial stresses (e.g. changes in relationships or work status), and aging. Akin to cognitive reserve, this model emphasizes that affective reserve is an active process, reflecting the brain’s ability to adapt when facing stressors.^1^ A capacity to regulate affective states implies that excessive or persistent negative emotions or moods underlie emotional challenges, in keeping with modern definitions of mood or anxiety disorders.^3,8^

**Figure 1.**
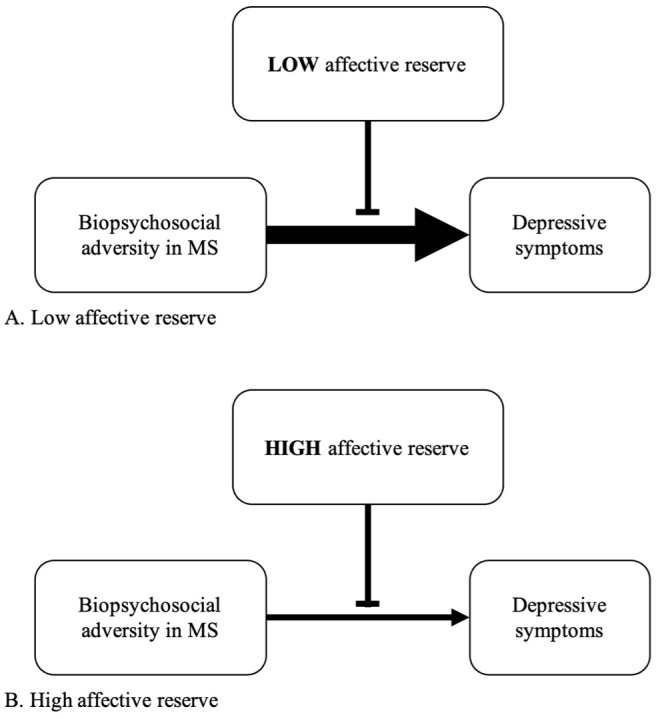
Conceptual model of how affective reserve moderates the relationship between biopsychosocial adversity and depressive symptom severity in MS. The thickness of arrows reflects the degree to which MS biopsychosocial adversity affects depressive symptoms. In this model, there is a stronger relationship between biopsychosocial adversity and depressive symptoms in individuals with a low affective reserve (i.e. thick arrow) compared to those with a high affective reserve (i.e. thin arrow).

To expand upon this concept, imagine two pwMS of similar ages, disease courses, Expanded Disability Status Scale scores, and psychosocial circumstances; one of whom is depressed and the other is not. What explains the difference? As depicted in Figure 1, this model hypothesizes that affective reserve moderates the relationship between biopsychosocial adversity and depressive symptoms in pwMS. Introducing the concept of affective reserve is important for a few reasons. As previously noted, depression is associated with substantial morbidity and mortality among pwMS.^3^ It is vital to identify potentially modifiable contributors to this risk, of which affective reserve may be one. In addition, the awareness that depression is more common in pwMS compared to the general population could foster therapeutic nihilism among patients and clinicians.^3^ Recognizing affective reserve offers hope that depression is not an inevitable outcome of severe biopsychosocial adversity in MS.

## Measures of affective reserve

Thus far, a valid and reliable measure of affective reserve in pwMS is not available. In the meantime, surrogate measures of affective reserve may include existing questionnaires that assess personality traits (e.g. low neuroticism), coping styles (e.g. problem-focused coping), or resilience. However, the use of these measures is complicated by potential bidirectional relationships between psychosocial factors and depression.^3^ Furthermore, this model posits that affective reserve is modifiable, and questionnaires to measure this construct must be sensitive to change. The complexity of measuring affective reserve highlights the need for the careful development of a dedicated index. While a genetic diathesis could be considered in the future, current evidence does not robustly support that a family history of depression predisposes a person with MS to depression.^3^

## Enhancing affective reserve

Psychotherapy could be one potential strategy to boost a person’s affective reserve. Cognitive-behavioral therapy or mindfulness-based interventions are evidence-based therapies with demonstrated efficacy in improving depressive symptoms in pwMS.^3^ These modalities involve teaching participants skills to adjust thoughts and behaviors, or to foster non-judgmental awareness of thoughts and feelings.^3^ A skills-focused treatment (in comparison to emotion-focused care) may be especially important for altering coping styles.^3^ In addition to mitigating stress, anxiety, or depressive symptoms,^3,10^ psychotherapy can affect the development of MS-related neuroimaging abnormalities.^10^ In a 24 week randomized controlled trial, an MS-tailored stress management program reduced negative life events and the accumulation of new gadolinium-enhancing or T2 lesions in pwMS compared to a waitlist control group.^10^ Although benefits were not maintained during the post-treatment follow-up period, these findings suggest that interventions targeting affective reserve could feedback to mitigate MS-related biopsychosocial adversity. Future randomized controlled trials of psychotherapy in pwMS should examine this possibility.

## Model highlights

The proposed model of affective reserve in MS has several strengths and the potential to influence clinical practice. Affective reserve complements existing research on cognitive and physical reserves, capturing an under-recognized dimension of reserve in pwMS. In addition, affective reserve is a simple concept with face validity, facilitating communication between pwMS, clinicians, and investigators in interdisciplinary investigations. In fact, this paper and our understanding of affective reserve reflect collaboration between researchers with clinical experience working with pwMS and who live with MS. Furthermore, it is often not possible to clinically disentangle whether depression is primarily due to MS disease activity, secondary to psychosocial stressors, or a combination of biological and psychosocial factors. Thus, this model is explicitly situated within a biopsychosocial framework, facilitating a broader understanding of the adversities experienced by pwMS and the potentially diverse approaches to bolster affective reserve. Notably, it remains unknown whether pharmacotherapy could bolster affective reserve in pwMS—an avenue for future research. In conjunction with cognitive and physical reserve, affective reserve supports a holistic understanding of reserve in pwMS.
